# Improved ex vivo fluorescence imaging of human head and neck cancer using the peptide tracer TPP-IRDye800 targeting membrane-bound Hsp70 on tumor cells

**DOI:** 10.1038/s41416-024-02872-8

**Published:** 2024-10-15

**Authors:** Katharina L. K. Holzmann, Johanna L. Wolf, Stefan Stangl, Philipp Lennartz, Atsuko Kasajima, Carolin Mogler, Bernhard Haller, Eva-Vanessa Ebert, Daniel Jira, Maren L. A. Lauterbach, Franziska von Meyer, Leonhard Stark, Leonie Mauch, Benedikt Schmidl, Barbara Wollenberg, Gabriele Multhoff, Markus Wirth

**Affiliations:** 1 Department of Otolaryngology - Head and Neck Surgery, Technical University of Munich (TUM), School of Medicine and Health, TUM University Hospital, Munich, Germany; 2 Department of Radiation Oncology and Central Institute for Translational Cancer Research (TranslaTUM), Technical University of Munich (TUM), School of Medicine and Health, TUM University Hospital, Munich, Germany; 3 Department of Nuclear Medicine and Central Institute for Translational Cancer Research (TranslaTUM), Technical University of Munich (TUM), School of Medicine and Health, TUM University Hospital, Munich, Germany; 4 Institute of Pathology, Technical University of Munich (TUM), School of Medicine and Health, TUM University Hospital, Munich, Germany; 5 Institute of AI and Informatics in Medicine, Technical University of Munich (TUM), School of Medicine and Health, TUM University Hospital, Munich, Germany

**Keywords:** Surgical oncology, Translational research

## Abstract

**Background:**

The primary goal of surgery in HNSCC is the complete resection of tumor cells with maximum preservation of normal tissue. The membrane Hsp70-targeting fluorescence labelled peptide TPP-IRDye800 represents a promising tool for real-time intraoperative tumor visualization, enabling the detection of true tumor margins, critical isles of high-grade dysplasia and LN metastases.

**Methods:**

Membrane Hsp70 (mHsp70) expression on HNSCC cell lines and primary HNSCC was determined by flow cytometry and fluorescence microscopy using FITC-conjugated mAb cmHsp70.1 and TPP. TPP-IRDye800 was sprayed on freshly resected tumor material of immunohistochemically confirmed HNSCC and LN metastases for tumor imaging. TBRs were compared using TPP-IRDye800 and Cetuximab-IRDye680, recognizing EGFR.

**Results:**

mHsp70 expressing HNSCC cells specifically bind and internalize TPP in vitro. The TBR (2.56 ± 0.39) and AUC [0.98 CI, 0.95–1.00 vs. 0.91 CI, 0.85–0.97] of TPP-IRDye800 on primary HNSCC was significantly higher than Cetuximab-IRDye680 (1.61 ± 0.39) (*p* = 0.0068) and TPP-IRDye800 provided a superior tumor delineation. Fluorescence imaging showed higher AUC values than a visual inspection by surgeons [0.97 CI, 0.94–1.00 vs. 0.92 CI, 0.88–0.97] (*p* = 0.048). LN metastases could be visualized using TPP-IRDye800. Real-time tissue delineation was confirmed using the clinically applied KARL-STORZ imaging system.

**Conclusion:**

TPP-IRDye800 is a promising fluorescence imaging probe for HNSCC.

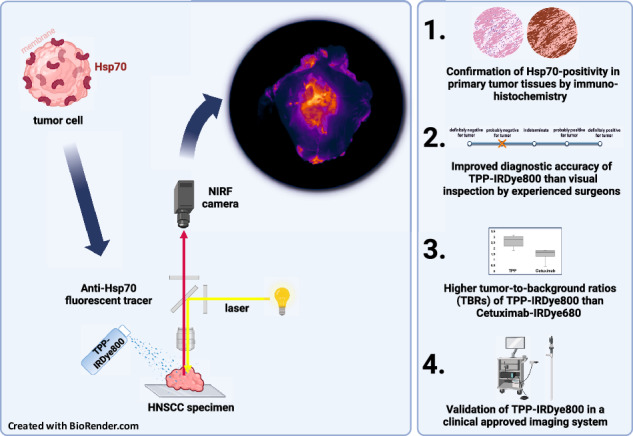

## Background

For the majority of solid tumors, including head and neck squamous cell carcinoma (HNSCC), resection is an elementary pillar for curative therapy. The aim of a successful cancer surgery is to localize and accurately delineate malignant from benign tissue to ensure cancer removal while minimizing damage to non-cancerous tissue. The importance of precise cancer surgery is evident, considering that incomplete tumor resection has a significant negative impact on local and regional recurrence, distant metastases, and overall survival [1, 2]. Nevertheless, preservation of adjacent healthy tissue is particularly important for treatments in the head and neck region in order to keep functional and aesthetic impairments as low as possible.

In daily clinical practice, surgeons mostly rely on visual inspection, manual palpation, and the analysis of frozen sections for a correct tissue differentiation [3]. The frequencies of inadequate resection margins reported in the literature are inconsistent and range from 30% up to 85% [4]. However, the unanimous conclusion from all studies is that the high rates of inadequate resection margins need to be improved [5].

A highly promising technology that has rapidly evolved over the last few years is real-time fluorescence-guided tumor imaging [6] which can support surgical navigation by identifying true tumor margins and even molecular alterations in or close to the tumor border, such as high-grade dysplasia which may not be visible by the naked eye. Given the easily accessible location of most head and neck tumors, it is not surprising that fluorescence imaging techniques are particularly mature in this tumor type and will likely be integrated in the operative workflow soon [7]. Since many tracers lack tumor cell specificity, the development of more effective tracers requires the identification of new tumor-specific targets.

One promising tracer is the tumor cell-penetrating peptide-based probe TPP [8], which recognizes an epitope of the stress-inducible molecular chaperone Heat shock protein 70 (Hsp70, HSPA1A) that is exclusively present on the cell surface of a large variety of human and mouse tumors, including head and neck [9–11]. A tumor-specific lipid composition [12] enables the location of Hsp70 on the plasma membrane of tumor, but not normal tissue cells. Therefore, mHsp70 provides a highly tumor-specific target for prognostic and therapeutic approaches and for diagnostic imaging [13–15].

The TPP tracer used in this study for the detection of mHsp70 is a 14-mer peptide that mimics the properties of the Hsp70 oligomerization domain [16]. Compared to larger antibody-based imaging probes small imaging molecules, such as peptides, have a higher tumor cell penetration efficacy, faster incorporation kinetic, a more efficient wash-out from normal tissues, lower immunogenic potential and a more favorable biodistribution [17–19].

In this pilot study, the in vitro binding characteristics and uptake kinetics of the fluorescence labelled TPP tracer were assessed in HNSCC cell lines and single cell suspensions of primary tumor material of HNSCC patients. Furthermore, we directly compared the tumor to background ratios (TBRs) and area under the curves (AUCs) of two topically administered tracers TPP and Cetuximab conjugated to the dyes IRDye800 and IRDye680 respectively, on the same HNSCC specimen to assess their tumor delineation capacity. The TPP tracer was also tested comparatively on lymph node (LN) metastases and negative LNs. Comparative immunohistochemistry was performed targeting Hsp70 and EGFR on respective tumor sections. To assess its potential clinical utility for real-time surgical decision making, the accuracy of the tumor delineation using the TPP tracer was compared with the visual inspection of unstained tumor tissues by five experienced head and neck surgeons.

## Materials and Methods

### Cells and cell culture

Two human HNSCC cell lines, CAL-27 (tongue, RRID:CVCL_1107, DSMZ, Braunschweig, Germany) and UD-SCC-5 (laryngeal, RRID:CVCL_L548, University of Düsseldorf, Germany) were cultured in high-glucose Dulbecco´s Eagle´s Minimum Essential Medium (DMEM) supplemented with 10% *v/v* heat-inactivated fetal bovine serum (FBS), 2 mM L-glutamine, 1 mM sodium-pyruvate and antibiotics (10,000 IU/ml penicillin and 10 mg/ml streptomycin), at 37 °C in 5% *v/v* CO_2_. Cells were passaged regularly twice a week. For the experiment the cells were used in the exponential growth phase and their viability was tested by trypan blue exclusion. All experiments were performed with mycoplasma-free cells. The cell lines have been authenticated using STR profiling within the last three years. All cell culture reagents were purchased from Sigma-Aldrich (St. Louis, MO, USA).

### Antibodies and peptides

The FITC-conjugated murine cmHsp70.1 monoclonal antibody (mAb, IgG1, multimmune GmbH, Munich, Germany) and an Hsp70-derived 14-mer peptide TKDNNLLGRFELSG (TPP) that has previously been demonstrated to bind to mHsp70 [16] were used. For flow cytometry and fluorescence microscopy studies, the TPP peptide was conjugated with FITC purchased from EMC Microcollections GmbH (Tübingen, Germany) and for the identification of tumor in freshly resected tissue of HNSCC patients the TPP peptide was conjugated to the near-infrared imaging probe IRDye800CW (Cambridge Research Biochemicals, Billingham, UK). Cetuximab (Erbitux^®^, RRID:AB_2459632, Merck, Darmstadt, Germany) which targets the epidermal growth factor receptor (EGFR), conjugated to IRDye680RD (IRDye680RD-NHS ester, LI-COR Biosciences, Lincoln, NE, USA), was used as a comparator for the identification of tumor tissue in resected tissue from patients with HNSCC.

### Fluorescence microscopy

Cells (10,000 cells/well) were cultured in 8-well chamber slides. After washing with phosphate-buffered saline (PBS), unlabelled cmHsp70.1 mAb was added as a blocking agent to reduce background staining. Following another washing step, the cells were left for 5 min at room temperature to allow viable cells to internalize the cmHsp70.1 mAb-labelled epitopes and re-express free epitopes. Subsequently, FITC-conjugated cmHsp70.1 mAb (100 µg/ml) or an IgG1 isotype-matched control antibody (mouse IgG1-FITC, Cat# 340755, RRID:AB_400127, BD Biosciences, Franklin Lakes, NJ, USA) were added. Cells were then incubated for either 8 min at 4 °C for cell surface staining of Hsp70 or 60 min at 37 °C for internalization. After washing three more times, cells were fixed with PBS containing 0.5% *w/v* paraformaldehyde (PFA) and embedded in 4’,6-diamidine-2-phenylindole (DAPI) solution (Vector Laboratories, Burlingame, CA, USA). The slides were imaged on the Zeiss Axio Observer Zl microscope (Carl Zeiss AG, Oberkochen, Germany). Images were acquired in brightfield, FITC and DAPI channels. Multicolor images were created by overlay.

### Flow cytometry

To determine the mHsp70 status, cells (0.2 × 10^6^) were incubated with FITC-labelled cmHsp70.1 mAb (2 µg/ml) or TPP (2 µg/ml) for 30 min at 4 °C in the dark. After washing with flow cytometry buffer (PBS containing 10% *v/v* heat inactivated FBS) cells were analyzed using a FACSCalibur™ flow cytometer (BD Biosciences). Flow cytometry was also performed with single-cell suspensions obtained from small tissue samples taken from the tumor area by an experienced pathologist. After mechanical disintegration of the tissue, which was performed as described previously [10], the cells were incubated with cmHsp70.1-FITC mAb and CD45-APC mAb (mouse IgG1, Cat# 555485, RRID:AB_398600, BD Biosciences). CD45 staining was used to distinguish peripheral blood leukocytes (major population) from CD45-negative, non-hematopoietic tumor cells.

In each flow cytometry analysis, propidium iodine (PI) was added prior to flow cytometric analysis in order to enable the exclusion of non-viable cells from the acquisition and analysis. An IgG1-FITC isotype-matched control antibody was used to evaluate nonspecific binding. The percentage of cells stained with the control antibody was subtracted from the percentage of mHsp70 positively stained cells.

### Internalization kinetics of cmHsp70.1 antibody and TPP

The targeting capacity of FITC-labelled cmHsp70.1 mAb and TPP was assessed by flow cytometry. For this, 200,000 cells were transferred to 1.5 ml tubes and washed with PBS. Each aliquot was incubated with the antibody or peptide at 4 °C or 37 °C for varying periods of time (0, 1, 2, 5, 10, 15, 20, 30, 60 to 120 min). After the indicated time points, the cells were transferred to tubes containing ice-cold PBS to stop internalization. After washing, cells were resuspended in flow cytometry buffer and analyzed as described above. Only viable (PI-) negative cells were gated and analyzed. The IgG1-FITC isotype-matched control antibody was included in all analyses.

### Patients

This study was approved by the Ethics Committee of the Klinikum rechts der Isar, Technical University Munich (TUM) School of Medicine and Health. All patients provided informed written consent 24 h before start of the study. A total of 11 patients with local surgical resection of HNSCC were included into the study. For the analysis of the tracer in lymph nodes one patient with Merkel cell carcinoma and one patient with adenoid cystic carcinoma who underwent primary tumor excision and neck dissection were included into the study. Patients were treated between April 2021 and Juni 2024 in the Department of Otorhinolaryngology at Klinikum rechts der Isar. Patients with varying tumor localizations and TNM-stages were included into the study.

### Ex vivo imaging

Immediately after excision, the tissue was covered for 5 min with a blocking solution (5% *w/v* milk powder and 0.5% *v/v* Triton-X-100 in PBS, all purchased from Sigma-Aldrich). For a direct comparison, the whole specimen was firstly sprayed with Cetuximab-IRDye680 (100 µg/ml) and then with TPP-IRDye800 (100 µg/ml). The concentration of 100 µg/ml for TPP was based on previous data indicating that this concentration provides an optimal TBR in vivo [14]. With respect to Cetuximab also a concentration of 100 µg/ml was chosen which is in line with a previous study with a topical application [20]. In vitro experiments displayed no overlap in the fluorescence signals with different wavelengths after a sequential application of Cetuximab-IRDye680 followed by TPP-IRDye800 on the same specimen. Specimens were incubated with both tracers for 5 min at room temperature (RT), followed by an intensive rinsing of the sample with PBS and 1% *v/v* Triton-X-100 before imaging. The IRDye600-dye was then excited with a 670 nm CW diode laser, and the emitted signal was passed through a 780/10 nm filter before being imaged with a back-illuminated EM CCD camera. IRDye800 was excited with a 780 nm CW diode laser, and the emitted signal was passed through an 800 nm long-pass filter. The camera captured an area of 2 × 2 cm. Images of larger specimen were stitched together using the ImageJ software (https://imagej.net/ij) [21]. In addition, lymph nodes with and without histologically proven metastases were imaged using TPP-IRDye800.

TBRs were determined by dividing the mean fluorescence value of cancerous regions of interest (ROIs) by the mean signal intensities of non-cancerous ROIs. The ROIs were selected randomly on the epithelial side of each resected specimen, maintaining a small distance from the electrosurgical cutting borders. For comparison of TPP and Cetuximab, identical ROIs on the same specimen were chosen. All fluorescence image analyses were performed using ImageJ software. Frozen sections of the tumors were obtained from pathologist as part of their intraoperative analysis. The glass slides with the frozen tissue were sprayed with TPP-IRDye680, and wide-field NIR macroscopic images were taken, as previously described. For microscopic imaging, the slides were embedded in DAPI solution and imaged using the 680-nm channel, as outlined in the fluorescence microscopy section.

### Evaluation of diagnostic accuracy

A three-step evaluation was performed for 12 selected ROIs. Initially, all ROIs were categorized using a binary system based on histopathology (1: tumor, 0: tumor-free) and validated by an experienced pathologist. Subsequently, the fluorescence intensity of either Hsp70- or EGFR-based imaging was quantified for each region, and a signal-to-background ratio was calculated by dividing each signal by the mean fluorescence value of the verified tumor-free ROIs within the same specimen. This facilitated a more effective intertumoral comparison.

Finally, five experienced otolaryngologists independently assessed the unstained ROIs visually using a five-point Likert scale: (1) indicating a definite absence of tumor; (2) likely absence; (3) indicating uncertainty; (4) suggesting probable presence; and (5) definite presence. The otolaryngologists are practicing in a certified University Hospital Center with a special focus on head and neck tumor surgery which assesses and treats a large number of tumor patients every year and are hence well trained. The surgeons’ evaluations were based on a white light image of the tumors, mirroring their intraoperative judgments. The inter-rater agreement was assessed and a receiver operator curve (ROC) was calculated using the conventional histologic assessment as gold standard.

### Histological assessment

Histological evaluation was performed on Hematoxylin and Eosin (H&E) stained slides. For the Hsp70 and EGFR immunohistochemistry staining, deparaffinized and rehydrated FFPE sections (4 µm) of the patient tumors were used and heated by microwaving in target retrieval buffer (pH 6) to unmask antibody epitopes. Nonspecific binding was blocked by incubating with a protein blocking solution (10% *v/v* rabbit serum in PBS and 1% *w/v* BSA) for 30 min. After each step, the sections were washed in PBS. An overnight incubation at 4 °C with the cmHsp70.1 mAb (dilution, 1:750 in PBS and 1% *w/v* BSA) or anti-EGFR mouse mAb (dilution, 1:100 in PBS and 1% *w/v* BSA, Cat# sc-373746, Santa Cruz Biotechnology, USA) was followed by another incubation with HRP-labelled rabbit anti-mouse secondary reagent (Cat# P0260, RRID:AB_2636929, Dako-Agilent, Santa Clara, CA, USA) for 2 h. Thereafter, a 3,3-diaminobenzidine (DAB + ) chromogen reaction, which was limited to exactly 5 min for all staining procedures was performed. Cell nuclei were counterstained with Hematoxylin. In a final step, all sections were embedded in Eukitt^®^ (Sigma-Aldrich) mounting medium. A reference section with a defined staining intensity was always examined as an internal control. Snapshot images of the stained sections were acquired using the Aperio Slide Scanner (Leica Biosystems, USA). Unless otherwise stated, the staining reagents were purchased by Agilent DAKO, USA. The expression levels of Hsp70 and EGFR in the tumor area were analyzed. To evaluate the staining intensity, tumor sections were categorized as very weak (value 0), weak (value 1), moderate (value 2), strong (value 3), and very strong (value 4). Adjacent normal mucosa was used as a control. For quantitative evaluation, the percentage of fields showing positively stained cells was considered. The values ranged from 0: < 1% positive stained tumor cells; 1: 1–25% positive tumor cells; 2: 25–50%; 3: 50–75% and 4: > 75%. The final expression level was then calculated by combining the qualitative and quantitative scores. Two independent investigators performed the assessment. All scores were blinded to the grading and the fluorescence intensities of each tumor sample.

### Clinical imaging system

The KARL STORZ IMAGE1 S™ Rubina® (KARL STORZ, Tuttlingen, Germany), a technology developed for NIR/ICG imaging, was evaluated for the detection of TPP-IRDye800. For this study the HOPKINS® NIR/ICG telescope (0°, 10 mm, 20 cm) was used in white light and NIR mode. Color videos and fluorescence images are displayed in real time. Via the IMAGE 1 S™ software menu control, different NIR/ICG visualization modes can be activated (overlay, intensity map, monochromatic). For comparison of the preclinical and clinical imaging systems, the exact same ROIs were selected and analyzed using ImageJ.

### Statistical analysis

Statistical analysis of the cell culture experiments as well as the comparison of TBR between TPP and Cetuximab was performed using Student´s t-test. Data are presented by mean ± SD. *P*-values ≤ 0.05 were considered significantly different between the compared groups. ROC analysis was used to evaluate the diagnostic performance of the fluorescence spray and the tumor assessment by surgeons compared to histopathology as gold standard. The differences between the calculated areas under the curve (AUC) were compared using the DeLong test. Analyses were performed using SigmaPlot software (Systat Software Inc, USA). The inter-rater agreement was assessed using a weighted κ coefficient for multiple raters with quadratic weights using the statistical software R [22] and its package irrCAC [23].

## Results

### Staining of Hsp70 on the membrane and in the cytosol of HNSCCs using the cmHsp70.1 antibody and TPP peptide

**Fig. 1:**
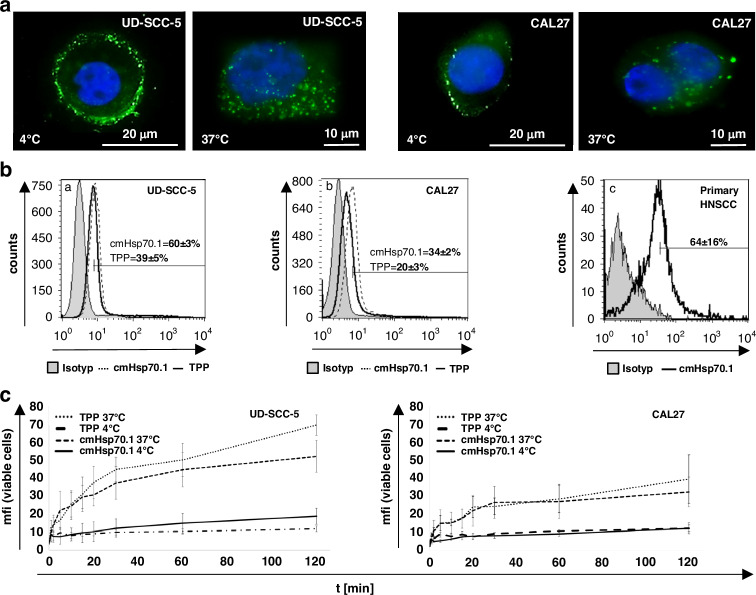
Analysis of Hsp70 expression of HNSCCs and their ability to internalize the TPP-tracer and cmHsp70.1 antibody. **a** Representative immunofluorescence images demonstrating the presence of Hsp70 on the cell surface and in the cytosol of UD-SCC-5 and CAL27. After incubation of the tumor cells with cmHsp70.1-FITC mAb (green) at 4 °C a typical ring-shaped cell surface staining pattern appeared (left upper graphs), whereas after an incubation at 37 °C Hsp70 staining was determined in the cytosol (right upper graphs). DAPI staining in blue visualizes the nucleus of the tumor cells. Scale bars: 10 µm, 20 µm. **b** Flow cytometric analysis of the mHsp70 expression after staining of viable tumor cell lines UD-SCC-5 (**a**), CAL27 (**b**) and single cell suspensions of primary tumor material derived from patients with HNSCC (**c**) with cmHsp70.1-FITC mAb (dotted lines) and TPP-FITC (solid lines) at 4 °C. The percentage of mHsp70 positively stained cells with both reagents was higher in UD-SCC-5 (60 ± 3% vs 39 ± 5%) compared to CAL27 cells (34 ± 2 vs 20 ± 3%); a relevant proportion of primary HNSCCs were positively stained with TPP-FITC (64 ± 16%). The data represent mean values ± SD of 3 independent experiments. The gray histograms illustrate the data of the staining with an isotype-matched control antibody. **c** In vitro uptake kinetics of cmHsp70.1-FITC and TPP-FITC into UD-SCC-5 (left) and CAL27 (right) tumor cell lines after an incubation period of 0 to 120 min at 4 °C (solid lines) and 37 °C (dotted lines). The uptake of both reagents was faster and stronger in UD-SCC-5 cells with a higher mHsp70 expression. The scale equals mean arbitrary units (au) of the fluorescence intensity (mfi).

Immunofluorescence stainings in Fig. 1a illustrate the presence of Hsp70 on the membrane of the UD-SCC-5 and CAL-27 HNSCC cell lines. After a 30 min incubation period of viable tumor cells with the FITC-labelled cmHsp70.1 monoclonal antibody (mAb) at 4 °C a typical ring-shaped surface staining could be seen in both tumor cell types (Fig. 1a, 4 °C). When tumor cells were incubated at 37 °C for 30 min a patchy intracellular staining pattern appeared which is caused by a translocation of mHsp70 stained with the FITC-cmHsp70.1 mAb into the cytosol (Fig. 1a, 37 °C). The intracellular and mHsp70 staining intensity appeared to be stronger in UD-SCC-5 cells compared to CAL-27 cells. Multiparameter flow cytometry using FITC-labelled cmHsp70.1 mAb and TPP peptide confirmed these findings and revealed a mHsp70 positive phenotype on 60 ± 3% and 39 ± 5% of UD-SCC-5, (Fig. 1b, a) and 34 ± 2% and 20 ± 3% of CAL-27 cells, respectively (Fig. 1b, b). In addition to the HNSCC cell lines, single cell suspensions derived from primary tumor material of a HNSCC patient stained with cmHsp70.1 mAb revealed a mHsp70 positivity on 64 ± 16% of the cells (Fig. 1b, c).

### Rapid internalization of TPP into HNSCCs via translocation of mHsp70 into the cytosol

Internalization kinetics of FITC-labelled TPP into tumor cells via a translocation of mHsp70 into the cytosol were performed to investigate the suitability of TPP as an imaging tracer. Flow cytometric analyses revealed a rapid and slightly faster internalization kinetics of the 1.5 kDa TPP peptide than the 150 kDa cmHsp70.1 mAb at 37 °C into the HNSCCs UD-SCC-5 (p = 0.093) and CAL-27 (*p* = 0.031) between 0 and 120 min (Fig. 1c). UD-SCC-5 cells with a higher mHsp70 expression density showed a faster uptake of FITC-labelled cmHsp70.1 mAb and TPP peptide, as determined by an increase in the mean fluorescence intensity (MFI) than CAL-27 cells. After 30 min the MFI of UD-SCC-5 cells incubated with cmHsp70.1 mAb and TPP increased 4.5-fold and 3.1-fold, respectively and 3.1-fold and 2.8-fold, respectively in CAL-27 cells. The intracellular increase in fluorescence was calculated as the quotient of the MFI after 30 min at 37 °C and at 4 °C, as described previously [24]. Since cmHsp70.1 mAb and TPP peptide are not internalized at 4 °C only surface bound Hsp70 is determined in the cytosol.

### Ex vivo fluorescence imaging using TPP-IRDye800 and Cetuximab-IRDye680 on resected tumor specimens from HNSCC patients

To evaluate the performance of TPP-IRDye800 for the delineation of tumor cells in tissue, the tracer was applied topically on freshly resected HNSCCs derived from a total of 11 different patients. The average age of the patients was 66.3 ± 11.3 years (Table 1). Patients with different TNM stages and tumor locations (oral cavity, oropharynx, tonsil) of mostly HPV-positive (*n* = 7) primary squamous cell carcinoma of the head and neck were included into the study (Table 1).

**Table 1:** Patient characteristics

**Age, mean (SD), years**		66.3 (11.3)
**Sex Male:Female**		9:2
**Site of lesion**		
	Oral cavity	4
	Oropharynx	3
	Tonsil	4
**History**		
	Primary	10
	Recurrence	1
**T-Staging**		
	T1	4
	T2	5
	T3	2
**N-Staging**		
	Nx	1
	N1	7
	N2a	1
	N2b	2
**HPV-Status**		
	Positive	7
	Negative	4
**Smoking history**		
	Yes	6
	No	4
	Unknown	1
**Alcohol consumption**		
	Yes	8
	No	2
	Unknown	1

**Fig. 2:**
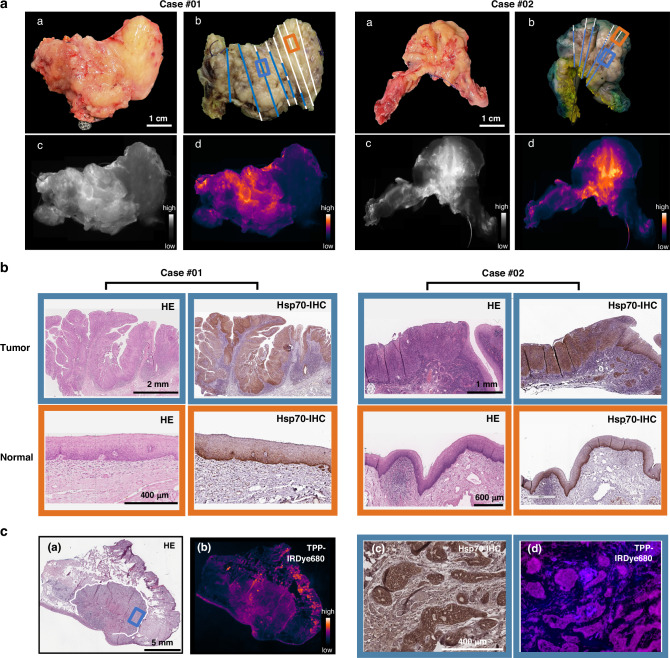
Ex vivo fluorescence imaging with TPP-IRDye800 and histological evaluation of specimen derived from patients with HNSCC. **a** Images of two squamous cell carcinoma cases (#01, #02) before (**a**) and after fixation (**b**). The fixed surgical specimens were sectioned as indicated by lines in different colors that represent different tissue types based on a pathohistological evaluation: blue, carcinoma; white, healthy tissue, dotted line; carcinoma in situ (CIS). Hsp70-based fluorescence imaging displayed in gray (**c**) and pseudo-colors (**d**). **b** Representative histological images: HE staining and immunohistochemistry (IHC) of Hsp70 in the tumor tissue (blue frame) and normal tissues (orange frame) that correspond to the marked areas in (**a**) (**b**). The data represent tumor of two different HNSCC patients. Scale bars: 1 mm, 2 mm, 400 µm, 600 µm. **c** Representative histological and microscopic images of the tissue from a patient with a carcinoma of the lip: HE staining is shown in (**a**), with the blue square indicating the region magnified in (**c**) and (**d**). A wide-field NIR image of the frozen tissue section after TPP-IRDye680 spray application is shown in (**b**). The tumor area (blue frame) is further analyzed with microscopic images of positive Hsp70 staining by immunohistochemistry (Hsp70-IHC) (**c**) and fluorescence microscopy using TPP-IRDye680 (**d**). Scale bars: 5 mm (**a**), 400 µm (**c**).

In all imaged cases, a strong signal enhancement of TPP-IRDye800 was detected within the tumor which could be clearly distinguished from the surrounding healthy tissue. In Fig. 2a two representative cases (case #01 and #02) before (*a*) and after (*b*) formalin fixation as well as Hsp70-based fluorescence imaging in gray scale (*c*) and in pseudo-colors (*d*) are illustrated. A histopathological assessment of the Hsp70 positivity in specimens of the same tumor tissues (Tumor, case #01 and #02) and the corresponding normal tissues (Normal) confirmed the tumor-specificity of the tracer (Fig. 2b). The mapping of the tumor region in the resected specimen was based on H&E and Hsp70-IHC sections and matched to fluorescence signals on the epithelial surface. Fluorescence imaging of a malignant fresh frozen tissue section after TPP-IRdye680 spray-application also revealed a distinct fluorescence signal (Fig. 2c, b) within the tumor area which was identified by H&E staining (Fig. 2c, a). On a cellular level, the tracer uptake was specific to tumor cells and correlated with an Hsp70 positive staining, as shown by immunohistochemistry (Fig. 2c, c) and fluorescence microscopy (Fig. 2c, d). Glandular cells exhibited an uptake of TPP (Fig. 2c, d), which could be attributed to the finding that Hsp70 is expressed in glandular intermediate filaments [25].

**Fig. 3:**
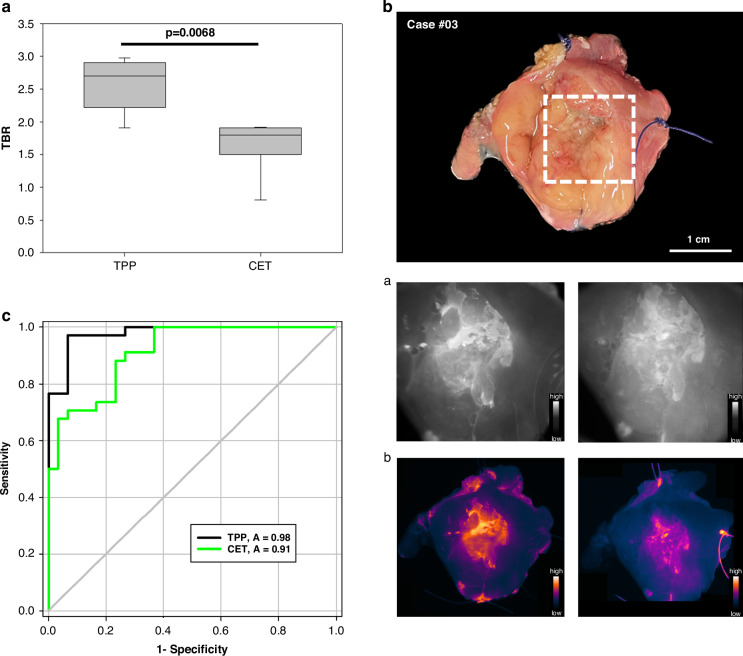
Comparison of the Tumor to background ratios (TBR) of TPP-IRDye800 versus Cetuximab-IRDye680. **a** Significantly higher TBRs (*p* = 0.0068) were detected for TPP-IRDye800 compared to Cetuximab-IRDye680. Data of 7 different HNSCC patients are shown as box plots. **b** Example of one representative tumor sample after sequential staining with TPP-IRDye800 and Cetuximab-IRDye680. The fluorescence images (**a**) show a less diffuse signal enhancement within the tumor and a clearer demarcation of the tumor to the surrounding healthy tissue for TPP-IRDye800 (left) compared to Cetuximab-IRDye680 (right). The pseudo-color overlay (**b**) reveals a stronger signal accumulation (yellow color code) in the tumor area for TPP-IRDye800 (left) compared to Cetuximab-IRDye680. Signal intensity scales are displayed in each image. **c** ROC curves were generated to compare diagnostic accuracy of fluorescence imaging using TPP-IRDye800 (black line) and Cetuximab-IRDye680 (green line). The AUC values of 0.98 (95% CI, 0.95–1.00) for TPP and 0.91 (95% CI, 0.85–0.97) for Cetuximab show a statistically significant difference (*p* = 0.025) for the ROIs of 6 patient samples.

A comparison of the TBRs after sequential staining with Cetuximab-IRDye680 and TPP-IRDye800 of the same tumor specimen revealed significantly higher TBRs for TPP (2.56 ± 0.39) than for Cetuximab (1.61 ± 0.39) (*p* = 0.0068) (Fig. 3a). The staining pattern of the tumor area with Cetuximab-IRDye680 was more diffuse and blurry (Fig. 3b, a) than that of TPP-IRDye800 which shows a clear demarcation of the tumor to the surrounding normal tissue (Fig. 3b, b). A comparison of the TBRs from 64 ROIs imaged with TPP-IRDye800 and Cetuximab-IRDye680 revealed significant differences with AUC values of 0.98 (95% CI, 0.95–1.00) for TPP and 0.91 (95% CI, 0.85–0.97) for Cetuximab (*p* = 0.025) (Fig. 3c).

Due to the necessity of an immediate pathological processing of the tumor specimen regarding further surgical decisions, time restrictions allowed the imaging with both fluorescence tracers, Cetuximab-IRDye680 and TPP-IRDye800, only in 7 of the 11 specimens, while the remaining 4 specimen were imaged only with TPP-IRDye800.

### Differences in the IHC staining intensities of Hsp70 and EGFR in tumor and normal tissues of HNSCC patients

**Fig. 4:**
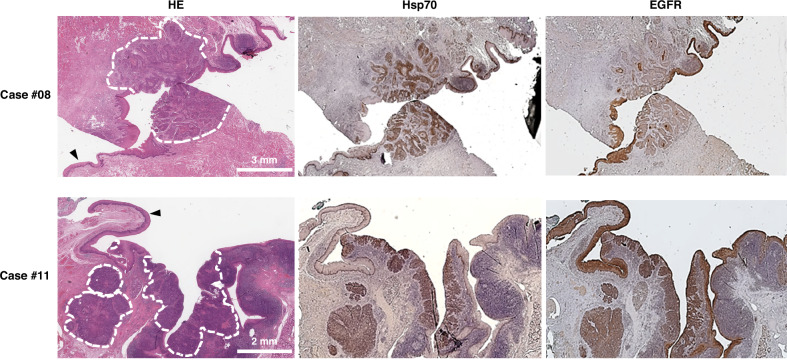
Comparison of the IHC staining intensities of Hsp70 and EGFR. Histological images of tissue sections from two patients with HNSCC. HE staining (left), immunohistochemical staining using cmHsp70.1 mAb (middle) and EGFR mAb (right). Dotted lines mark the pathological confirmed tumor areas and arrows indicate healthy epithelial tissue. Scale bars: 2 mm, 3 mm.

Immunohistochemical staining with the cmHsp70.1 mAb revealed a clear difference between tumor and normal tissues, as seen in two representative cases #08 and #11 (Fig. 4). Since all nucleated cells contain Hsp70 in the cytosol but not on the cell surface [26], a very weak intracellular staining pattern of Hsp70 (score 0) was also observed in basal layer of healthy epithelial tissue (Fig. 4). The Hsp70 expression in tumor areas varied and ranged from very low to very high. Carcinoma in situ (CIS) lesions (*n* = 2) were also found to be positively stained with the TPP tracer, but not with Cetuximab.

IHC staining with anti-EGFR mAb revealed a low to very high expression of EGFR within the tumor areas. However, due to a very strong staining intensity of EGFR in the normal epithelium, a differentiation between healthy and tumor tissue was not always possible (Fig. 4, case #11).

### Comparative diagnostic accuracy of the Hsp70-based fluorescence imaging and traditional surgeons’ assessment

**Fig. 5:**
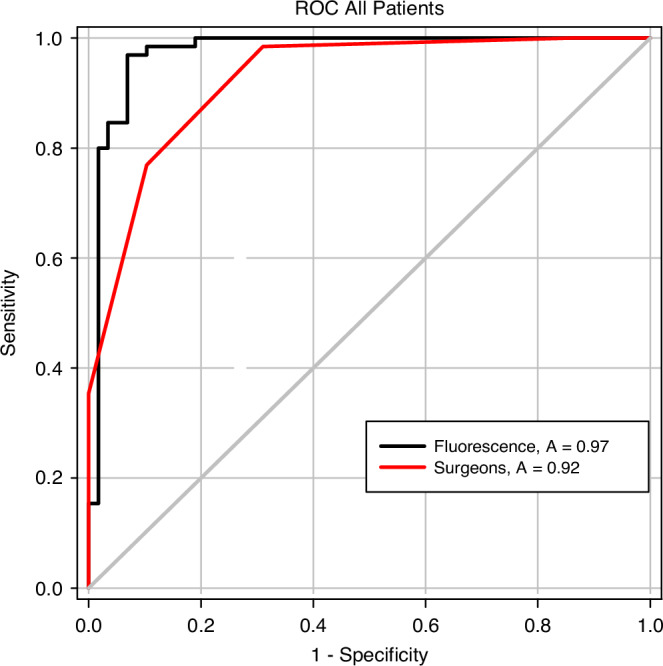
Evaluation of diagnostic accuracy of the Hsp70-based fluorescence imaging vs. traditional surgeons‘ assessment. ROC curves were generated for the evaluation of diagnostic accuracy of fluorescence imaging (black line) and visual assessment by surgeons (red line). The AUC values of 0.97 (95% CI, 0.94–1.00) for fluorescence imaging and 0.92 (95% CI, 0.88–0.97) show a statistically significant difference (*p* = 0.048) for the ROIs of all patient samples (*n* = 11).

To evaluate diagnostic accuracy, fluorescence imaging using TPP-IRDye800 was compared to a visual tissue inspection by qualified surgeons. The classification of selected ROIs of tumor tissues into cancerous and tumor-free by the two methods was compared to a classical histopathologic evaluation as the gold standard. A total of 123 ROIs were analyzed. Nine of the previously selected ROIs could not be evaluated due to the absence of processed histological material. The AUC values of 0.97 (95% CI, 0.94–1.00) for the topical application of the TPP tracer and 0.92 (95% CI, 0.88–0.97) for the visual assessment by 5 experienced surgeons (Fig. 5) differed statistically significantly (*p* = 0.048). For agreement between the five raters, a κ coefficient of 0.63 (95% confidence interval 0.56 to 0.71) was determined, indicating a substantial agreement between the ratings [27]. The optimal TBR threshold, at which a region of interest (ROI) can be identified as a tumor lesion using fluorescence imaging, was determined through the calculation of the Youden Index. The highest Youden Index (0.90) was achieved at a TBR of 1.75.

### Ex vivo fluorescence imaging using TPP-IRDye800 on Hsp70 negative lymph nodes and Hsp70 positive lymph node metastases

**Fig. 6:**
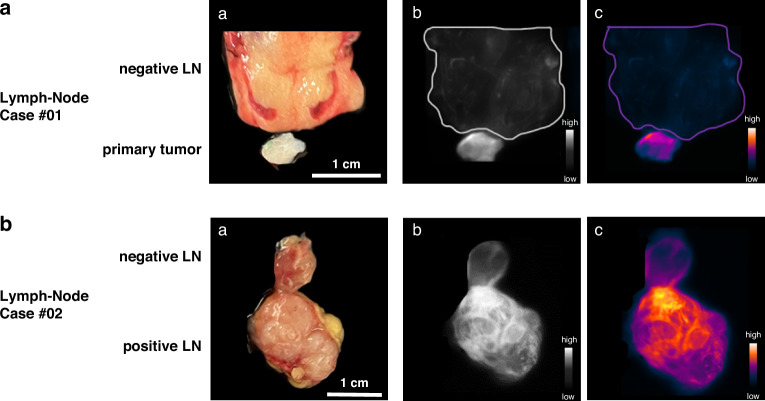
Identification of metastatic lymph nodes (LN) using Hsp70-based fluorescence imaging. Images of Hsp70-negative and Hsp70-positive lymph nodes (LNs) and a tumor fragment from two patients after primary tumor excision and neck dissection. **a** Comparison between an Hsp70-negative LN and an Hsp70-positive fragment of the primary tumor. Fluorescence imaging showed a signal-to-background ratio (SBR) of 3.15 for the primary tumor. **b** Comparison between an Hsp70-negative LN and an Hsp70-positive LN metastasis (SBR = 1.97). White light images (**a**) and Hsp70-based fluorescence images displayed in gray scale (**b**), and pseudo-color (**c**) are shown. Scale bar: 1 cm

To address the question whether Hsp70-based imaging can also detect malignancies in lymph nodes (LNs), TPP-IRDye800 was sprayed on LN with and without histologically confirmed metastases and a fragment of the primary tumor (Fig. 6a, b). A comparison of a patient´s metastasis free LN with a fragment of the primary carcinoma (adenocystic carcinoma, Fig. 6a) clearly revealed a strong positive staining with TPP-IRDye800 in the malignant primary tumor fragment (SBR = 3.15) but not in the negative LN as determined by gray scale (Fig. 6a, b) and pseudo-color staining (Fig. 6a, c). When comparing an Hsp70 positive and negative LN from another patient with Merkel cell carcinoma (Fig. 6b, a), the lymphatic metastasis exhibited a stronger fluorescence signal than the negative LN (SBR = 1.97) (Fig. 6b b, c).

### Clinical imaging system detects and delineates HNSCc in resected tissues stained with TPP-IRDye800

**Fig. 7:**
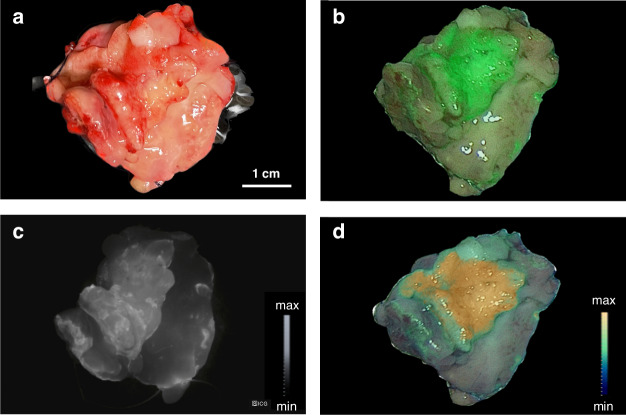
Hsp70-based fluorescence imaging using a Clinical Imaging System. Imaging of the resected tissue of a HNSCC patient using the clinically applied KARL STORZ IMAGE1 S™ Rubina® system. Fluorescence images were displayed under white light (**a**) and near infrared fluorescence (NIR), displayed are: overlay (**b**); gray scale (**c**) and intensity map (**d**). Picture (**d**) is taken from the real-time fluorescence imaging video which is shown in the supplementary material (Supplementary File 1).

A dilution series of TPP-IRDye800 ranging from 0.5 µg/ml to 200 µg/ml was used for the imaging with the KARL STORZ IMAGE1 S™ Rubina® system. The fluorescence tracer signal was adequately detected up to a concentration of 5 µg/ml. The imaging technology was also evaluated on patient specimen which was stained with the TPP-IRDye800 tracer in an overlay (Fig. 7a). Using the systems different overlay options (Fig. 7b), the tumor could be visualized specifically and with sufficient contrast to healthy tissue, as determined by near infrared fluorescence imaging in gray scale (Fig. 7c) and intensity map with the tumor signal reaching the highest color coding on the intensity scale of the device (yellow) (Fig. 7d). When the channel was switched to a monochromatic view (Fig. 7c), a TBR of 2.21 ± 0.46 was reached.

The system enables real-time presentation of the fluorescence overlay required for the use of real-time fluorescence-guided surgery, as demonstrated in a supplementary movie (Supplementary File 1). Congruent signal enhancement was subsequently observed with the preclinical imaging system with a signal to background ratio of 2.43 ± 0.56.

The specificity of the topically applied TPP-IRDye800 tracer could be confirmed in a tumor mouse model showing a strong fluorescence signal within the tumor with a TBR of 2.38, but no significant staining in normal mouse tissues such as skin, spleen and intestine (Supplementary File 2). The mean penetration depth after topical application of TPP-IRDye800 on human HNSCC tissue for 5 min at room temperature was 0.72 ± 0.03 mm indicates a rapid uptake of the tracer into tumor tissue (Supplementary File 3).

## Discussion

Over the past decade, optical fluorescence imaging has emerged as a promising technique for enhancing the precision of tumor resection. Therefore, a growing interest in targeted fluorescence imaging probes binding tumor-associated factors has evolved [28]. Tumor-specificity and high signal-to-background ratios are essential requirements for a successful fluorescent imaging tracer. Both properties were demonstrated for the imaging agent TPP-IRDye800 in this study, thereby enabling an effective detection and delineation of HNSCC tissue from the surrounding normal epithelial tissue. A real-time tumor delineation of HNSCC was confirmed with a clinically approved NIR-imaging device which enables a rapid clinical translation.

The tumor-specificity of TPP-IRDye800 can be explained by the exclusive expression of the target molecule, mHsp70 on the surface of a large variety of different and highly aggressive tumor cells but not on the membrane of corresponding normal cells [11, 29]. The rapid uptake of the imaging agent into tumor cells after binding to the target molecule facilitates a fast intracellular accumulation and high TBRs. Previous studies, along with data provided here, have confirmed a rapid uptake of fluorescence-labelled cmHsp70.1 mAb and TPP peptide into tumor cells in vitro at 37 °C. This phenomenon can be attributed to a rapid turnover rate (approximately 15 min) of mHsp70 into the cytosol via a non-classical endo-lysosomal pathway [19]. A high membrane expression density of Hsp70 on tumor cells, as detected on UD-SCC-5 cells, is associated with a more rapid internalization kinetics of mHsp70-targeting molecules such as the cmHsp70.1 mAb and TPP into HNSCC cells. This finding is consistent with previous results in other tumor types [16] .

Especially in the field of head and neck cancer, fluorescence imaging techniques are close to clinical application [7]. Research on the FDA-approved EGFR antibodies Cetuximab and Panitumumab for targeted fluorescence imaging are most advanced with ongoing phase 2 clinical trials [30, 31]. It was for that reason that the imaging performance of the mHsp70-targeting tracer TPP was comparatively analyzed to that of the established EGFR-targeting tracer Cetuximab. In our setting, we showed that the application of TPP-IRDye800 to patient specimens yielded significantly higher TBRs than Cetuximab-IRDye680. A more diffuse and blurry staining pattern with a less clear discrimination of the tumor to the surrounding normal tissue was seen with Cetuximab-IRDye680 compared to TPP-IRDye800. This could be explained by the fact that although EGFR is overexpressed in 90% of HNSCC [32], it is not exclusively expressed on the surface of tumor cells, but is also present at lower levels on normal cells [33]. Although IHC staining of EGFR revealed a high expression density in the tumor areas, a clear differentiation between healthy and tumor tissue was difficult because of a strong staining of normal epithelial cells as well as metaplastic and dysplastic lesions in the tumor environment [34].

An interesting application potential of fluorescence-guided surgery is its ability to identify even microscopic inconspicuous cancer-associated genetic alterations in adjacent tissues to the tumor. They pose a significant challenge in surgery as they may elude detection by surgeons [35–37]. These malignant alterations, in particular high-grade dysplasia, need to be resected, as they harbor a high risk to progress to an invasive cancer [38, 39]. Our study demonstrated for the first time that TPP-IRDye800 has the potential to detect high-grade dysplastic lesions (CIS) in two HNSCC patients and might help to detect LN metastases.

The applied amount of TPP-IRDye800 and Cetuximab-IRDye680 in the ex vivo spray application was aligned according to an identical concentration of 100 µg/ml. Previous studies have shown that this concentration results in optimal TBRs of both tracers after spray application [14, 20].

To compare identical ROIs within the tumor specimen, the tracers were conjugated with two different dyes. Since both dyes belong to the NIR spectrum with correspondingly long wavelengths, their biophysical properties, such as autofluorescence, are comparable. However, due to the marginally lower emission wavelength of IRDye680, there is a possibility of a slightly reduced tissue penetration capacity and an increased scattering in deep tissue. In our experimental setup, in which both tracers were applied topically, scattering problems in deeper tissue layers play only a minor role. The topical application of tracers offers remarkable advantages, notably reducing the risk of side effects and eliminating the need for additional hospital visits for the injection of the fluorescent agent prior to surgery. The specificity of TPP-IRDye800 after topical application could also be confirmed in a mouse model. Various organs such as skin, spleen and intestine were not stained with the tracer after topical application on resected organs, while the tumor demonstrated a strong fluorescence signal (Supplementary File 2). Nevertheless, a topical application is only suitable for assessing the exposed surface layers [40], and depending on the depth of penetration of the fluorescence-labelled tracer into the tissue a repeated spray application of the tracer might be necessary. Although the mean penetration depth of the tracer TPP-IRDye800 into human tumor tissue after topical application for 5 min at room temperature was 0.72 ± 0.03 mm (Supplementary File 3), a bleeding situs might hinder a homogenous distribution of the imaging agent and therefore might result in false negative imaging results.

Another limitation of a topical administration of fluorescence-labelled tracers is the non-specific accumulation of the tracer at the cutting areas of specimen after electrosurgical resection. These signal artefacts occurred with both tracers TPP-IRDye800, CetuximabIRDye680, as well as albumin, and also have been described for γ-glutamyl-hydroxymethyl rhodamine green (gGlu-HMRG) [41], a fluorescent targeting agent that can be enzymatically activated with γ-glutamyl-transpeptidase (GGT). In this study using gGlu-HMRG to detect superficial HNSCC, the authors suggested that the signals are caused by autofluorescence [41] or denatured proteins [42] as a result from electrosurgery-induced burning. Apart from signal artefacts, time and temperature issues can also limit the effectiveness of the topical ex vivo spray technique. Due to the required incubation periods for both tracers the whole imaging process takes about 20–30 min. Additionally, the incubation of the tracer was performed at room temperature and not at 37 °C which might negatively affect the optimal internalization kinetics of TPP. Therefore, after successful finalizing toxicity studies it is planned to inject the TPP tracer intravenously (i.v.) into HNSCC patients. As already shown for Cetuximab-800CW [20, 43] an i.v. injection of the TPP tracer might yield higher TBRs than a topical application, and a non-specific tracer accumulation on the cutting areas by electrosurgery could be avoided.

Hsp70 is involved in most cancer hallmarks, including the prevention of apoptosis, promotion of replicative immortality, and activation of invasion and metastasis [44]. Therefore, elevated levels of intracellular Hsp70 are associated with poor prognosis [45–47]. Vesicular Hsp70 in the blood of tumor patients which is actively released by viable tumor cells expressing high levels of Hsp70 in the cytosol and on the membrane [48] can reflect viable tumor mass and predict therapeutic response [49]. Therefore, we propose to measure circulating vesicular Hsp70 levels as a companion diagnostic to identify patients with mHsp70 positive tumors and those that most likely benefit from a TPP-based tumor imaging approach.

In conclusion, TPP-IRDye800 is a promising fluorescence tracer for a more precise resection of tumors and CIS in the head and neck area. The findings of this pilot study pave the way for an i.v. injection of the TPP tracer in larger clinical trials.

## Supplementary information

Supplementary file 1 to 3

Supplementary file 1

## Data Availability

All relevant data related to this study are included within the article or in the supplementary materials. Further data will be provided upon reasonable request to the corresponding author.
