# Supplementary material for: Improved ex vivo fluorescence imaging of human head and neck cancer using the peptide tracer TPP-IRDye800 targeting membrane-bound Hsp70 on tumor cells
Source: Br J Cancer. 2024 Oct 15;131(11):1814–24. doi: 10.1038/s41416-024-02872-8 (PMC11589746; doi:10.1038/s41416-024-02872-8)
Supplement: Supplementary file 1 — to 3 [file 41416_2024_2872_MOESM1_ESM.pdf]

Supplementary File 1. Real-Time Fluorescence Imaging using Clinical Imaging System (MOV 1)

Video of excised patient tissue after spray application of TPP-IRDye800. First, the tissue is displayed in white light mode, then the system is switched to real-time fluorescence overlay. The tumor tissue shows a high signal accumulation in yellow which represents the highest color coding on the intensity scale of the device. The tumor tissue can be clearly distinguished from the healthy surrounding tissue.

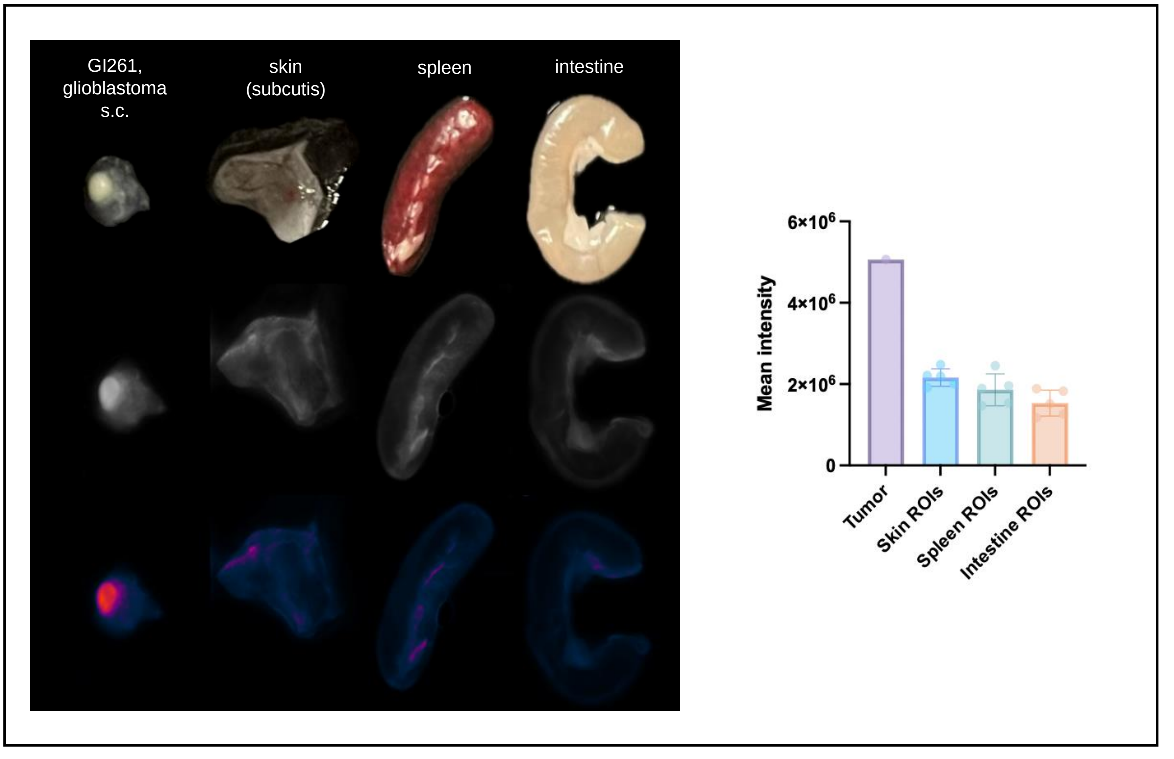

Supplementary File 2. Staining Specificity of Topically Applied TPP-IRDye800 in a Tumor Mouse Model

TPP-IRDye800 was applied as a spray to various organs of a mouse (skin, spleen, intestine), including a subcutaneously (s.c.) injected brain tumor (Gl261), after tissue extraction. The tumor exhibited a strong fluorescent signal, with a TBR of 2.38. The normal mouse tissues showed no significant staining with the TPP-IRDye800 tracer.

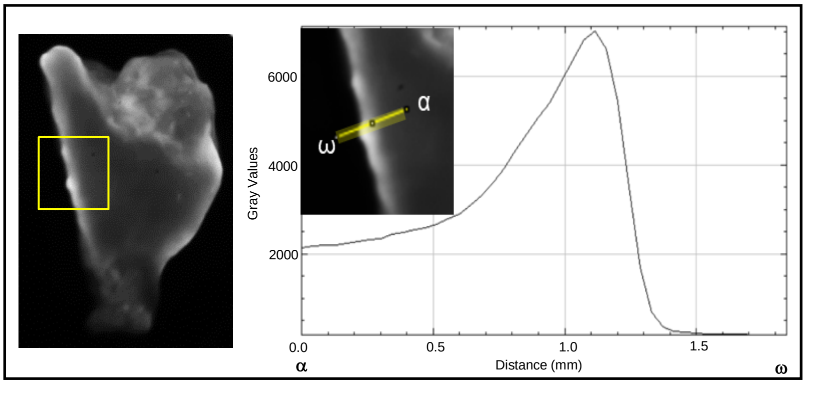

Supplementary File 3. Quantification of the Penetration Depth of the TPP-IRDye800 into Tumor Tissue

After an incubation of human HNSCC tissues with TPP-IRDye800 for 5 minutes at room temperature the mean penetration depth was 0.72±0.03 mm. The histogram shows gray values along the line shown in the inlay (α to ω).
